# Theoretical Analysis of Contact Angle and Contact Angle Hysteresis of Wenzel Drops on Superhydrophobic Surfaces

**DOI:** 10.3390/nano14231978

**Published:** 2024-12-09

**Authors:** Yufeng Li, Junyan Liu, Jialong Dong, Yufeng Du, Jinchun Han, Yuanyuan Niu

**Affiliations:** 1 College of Electrical and Power Engineering, Taiyuan University of Technology, Taiyuan 030024, Chinahanjc1998@163.com (J.H.); 2 SEDIN Engineering Co., Ltd., Taiyuan 030000, China; 3 Inner Mongolia Electric Power (Group) Co., Ltd., Ordos Power Supply Branch, Ordos 017000, China

**Keywords:** contact angle, contact angle hysteresis, micro/nanostructured superhydrophobic surfaces, Wenzel state

## Abstract

Although understanding the wetting behavior of solid surfaces is crucial for numerous engineering applications, the mechanisms driving the motion of Wenzel drops on rough surfaces remain incompletely clarified. In this study, the contact angle and contact angle hysteresis of Wenzel drops on superhydrophobic surfaces are investigated from a thermodynamic perspective. The free energy of the system is theoretically analyzed, thereby determining the equilibrium contact angle. Based on the sessile drop method, the relationship between the free energy barrier and the drop volume is calculated quantitatively, enabling the determination of advancing and receding contact angles under zero free energy barrier conditions. The theoretical calculations agree well with the experimental data. These findings enhance the understanding of the interfacial interactions between Wenzel drops and superhydrophobic surfaces.

## 1. Introduction

The wetting properties of superhydrophobic surfaces featuring micro- and nanostructures have been the focus of considerable scientific investigation in recent years [1,2,3], driven by many potential applications across various fields, such as self-cleaning materials [4,5], microfluidic devices [6], anti-icing surfaces [7,8] and anti-flashover coatings [9,10]. A critical parameter characterizing the wettability exhibited by these superhydrophobic surfaces is contact angle hysteresis [11,12,13]. This parameter allows for a detailed analysis of the dynamic behavior of water drops on solid surfaces [11,12]. Normally, a lower contact angle hysteresis is a prerequisite for the widespread application of superhydrophobic surfaces [5,10,14,15].

The underlying mechanisms governing the wetting behavior on these surfaces are typically attributed to the synergistic effect of microscopic or nanoscopic surface texture combined with coatings designed to lower surface energy [5,10,14]. Some experimental results indicate that the dynamics of the contact line significantly influence contact angle hysteresis on solid surfaces. Paxson et al. utilized environmental scanning electron microscopy to observe advancing contact line pinning on micropillared surfaces, which resulted in advancing contact angles reaching 180° [16]. Schellenberger et al. used an inverted laser-scanning confocal microscope to observe similar phenomena [17]. Jiang et al. employed reflective interference contrast microscopy to investigate the effects of micropillars and micropores on contact line motion, elucidating the different adhesion behaviors of water drops on these distinct surface structures [18]. Several theoretical models have been proposed to analyze contact angle hysteresis on low-adhesion superhydrophobic surfaces. Shi et al. developed a methodology for contact angle hysteresis on micropillar and micro-channel structured superhydrophobic surfaces by analyzing the transitional states during the receding and advancing processes of water drops [19]. Yao et al. proposed a model to elucidate the detachment behavior of water drops from micropillared superhydrophobic surfaces, utilizing experimentally measured lateral retention force [20]. Li theoretically calculated the contact angle hysteresis of superhydrophobic surfaces by analyzing the changes in the free energy barrier during variations in drop volume [21]. Zhu et al. constructed a theoretical model for contact angle hysteresis based on energy conservation, establishing the relationship between gravitational work and adhesion work during drop rolling [22]. These theoretical models agree well with experimental results, significantly advancing the understanding of contact angle hysteresis.

Despite the numerous practical applications of low-adhesion superhydrophobic surfaces, certain applications, such as drop transportation [23] or chemical microreactions [24], may benefit from surfaces exhibiting higher contact angle hysteresis [18]. For superhydrophobic surfaces, the state in which a drop is in complete contact with a rough surface is generally regarded as the Wenzel state (with the corresponding drop termed a Wenzel drop) [25,26]. In the Wenzel state, the adhesion between the drop and the surface significantly increases [13]. Therefore, there is a critical need to clarify the underlying physical mechanisms governing contact angle hysteresis of Wenzel drops on micro- and nanostructured surfaces. However, theoretical debates surrounding this issue persist. Traditionally, surface roughness has been considered a key factor influencing contact angle hysteresis behavior in the Wenzel state [26]. However, Gao et al.’s findings suggest that contact angle hysteresis is primarily driven by the interactions between surface microstructures at the drop boundary and the three-phase contact line [27]. Moreover, although the Wenzel equation implies that rough hydrophilic surfaces exhibit enhanced hydrophilicity compared to smooth hydrophilic surfaces [26], Forsberg et al.’s experimental results reveal that rough hydrophilic surfaces can exhibit advancing contact angles above 90° [28]. These apparent contradictions emphasize the need for further research into contact angle hysteresis on micro/nanostructured surfaces for the Wenzel state.

This study proposes a theoretical model to analyze the wettability for Wenzel drops on pillared surfaces. First, the free energy of the system was theoretically analyzed, allowing for the computation of the equilibrium contact angle. Subsequently, using the sessile drop method, a relationship between the free energy barrier and the volume of the Wenzel drop was established, thereby developing a theoretical model for numerical contact angle hysteresis calculations. Finally, experimental results from the literature were used to validate the theoretical model. The results indicate that the developed model can accurately predict contact angle hysteresis, providing a robust theoretical framework for understanding this phenomenon.

## 2. Theoretical Model

### 2.1. Free Energy of the Wenzel Wetting System

**Figure 1:**
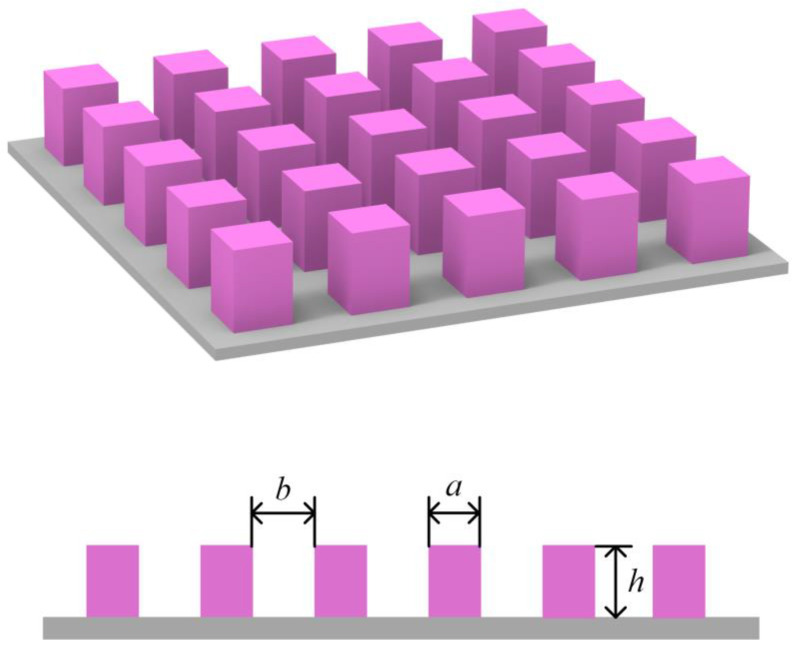
Illustration of a pillared surface.

Investigating the wetting behavior of Wenzel drops on superhydrophobic surfaces involves the use of a three-dimensional pillared surface, as depicted in Figure 1. This surface topology features pillars defined by their width (*a*), spacing (*b*), and height (*h*), which serve as structural parameters for thermodynamic analysis and the subsequent calculation of contact angle hysteresis.

**Figure 2:**
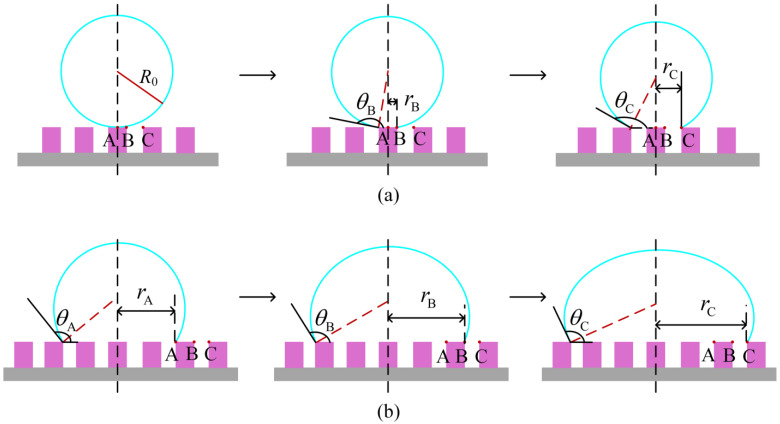
Illustration of a drop wetting a pillared surface. (**a**) Initial placement of the drop on the surface. (**b**) Continuous advancement of the contact line.

The system’s free energy is described by the equation [29]:

(1) $$F=\gamma_{\mathrm{sa}}S_{\mathrm{sa}}+\gamma_{\mathrm{sl}}S_{\mathrm{sl}}+\gamma_{\mathrm{la}}S_{\mathrm{la}}$$

in which the interfacial tensions are denoted by *γ*_sa_, *γ*_sl_, and *γ*_la_, corresponding to the solid–air, solid–liquid, and liquid–air interfaces, respectively. The surface areas associated with these interfaces are given by *S*_sa_, *S*_sl_, and *S*_la_. A drop, upon deposition on the pillared surface, initially presents a spherical shape, and makes initial contact at position A, as shown in Figure 2a. To minimize the system’s free energy, the contact line shifts its location and the drop’s shape changes [30]. The thermodynamic analysis of the free energy for the Wenzel state, when a drop wets the surface, is as follows. During the initial stage, with the movement of the contact line from position A to B (Figure 2a), the free energy change is simplified using Young’s equation [31,32] and is expressed as

(2) $$\frac{\Delta F_{A\to B}}{\gamma_{\mathrm{la}}}=\frac{2\pi r_{B}^{2}}{1+\cos\theta_{B}}-4\pi R_{0}^{2}-a^{2}\cos\theta_{Y}$$

where *θ*_Y_ is the intrinsic contact angle; *R*_0_ is the drop radius at position A; and *r*_B_ and *θ*_B_ are the base radius and the apparent contact angle at position B, respectively. The volume of the drop at positions A and B can also be obtained:

(3) $$V_{A}=\frac{4}{3}\pi R_{0}^{3}$$

(4) $$V_{B}=\frac{\pi r_{B}^{3}}{3\sin^{3}\theta_{B}}\left(\cos^{3}\theta_{B}-3\cos\theta_{B}+2\right)$$

As the contact line advances from position B to C, the free energy change and the volume of the drop is obtained:

(5) $$\frac{\Delta F_{B\to C}}{\gamma_{\mathrm{la}}}=\frac{2\pi r_{C}^{2}}{1+\cos\theta_{C}}-\frac{2\pi r_{B}^{2}}{1+\cos\theta_{B}}+2\pi r_{C}h-\left(\pi r_{C}^{2}+4ah-a^{2}\right)\cos\theta_{Y}$$

(6) $$V_{C}=\frac{\pi r_{C}^{3}}{3\sin^{3}\theta_{C}}\left(\cos^{3}\theta_{C}-3\cos\theta_{C}+2\right)+\left(\pi r_{C}^{2}-a^{2}\right)h$$

where *r*_C_ and *θ*_C_ are the base radius and the apparent contact angle at position C, respectively. Due to the conservation of mass, *V*_A_ should equal *V*_B_ and *V*_C_.

The pillared surface can be idealized as a repeating unit consisting of pillar spacing and width. Consequently, the system’s states at locations A, B, and C (Figure 2b) represent a typical scenario where the contact line moves continually through a series of pillar widths and spacings. Following the previous analysis, as a drop advances from A to B, the changes in free energy and the volume of the drop can be calculated using Equations (7) and (8):

(7) $$\frac{\Delta F_{A\to B}}{\gamma_{\mathrm{la}}}=\frac{2\pi r_{B}^{2}}{1+\cos\theta_{B}}-\frac{2\pi r_{A}^{2}}{1+\cos\theta_{A}}+\frac{2\pi br_{B}h}{a+b}-2\pi r_{A}h-\frac{2\pi r_{B}\left(a^{2}+ab+3ah\right)}{a+b}\cos\theta_{Y}$$

(8) $$V_{B}=\frac{\pi r_{B}^{3}}{3\sin^{3}\theta_{B}}\left(\cos^{3}\theta_{B}-3\cos\theta_{B}+2\right)+\pi h\left(r_{B}^{2}-a^{2}{\left(\frac{r_{B}+b}{a+b}\right)}^{2}\right)$$

As the drop continues to advance from B to C, the changes in free energy and the volume of the drop are derived from Equations (9) and (10):

(9) $$\frac{\Delta F_{B\to C}}{\gamma_{\mathrm{la}}}=\frac{2\pi r_{C}^{2}}{1+\cos\theta_{C}}-\frac{2\pi r_{B}^{2}}{1+\cos\theta_{B}}+2\pi r_{C}h-\frac{2\pi br_{B}h}{a+b}-\left(\pi r_{C}^{2}-\pi r_{B}^{2}+\frac{2\pi ar_{B}h}{a+b}\right)\cos\theta_{Y}$$

(10) $$V_{C}=\frac{\pi r_{C}^{3}}{3\sin^{3}\theta_{C}}\left(\cos^{3}\theta_{C}-3\cos\theta_{C}+2\right)+\pi r_{C}^{2}h\left(\frac{b^{2}+2ab}{{\left(a+b\right)}^{2}}\right)$$

Utilizing Equations (2)−(10), an iterative computational scheme for the free energy in the Wenzel state can be developed.

### 2.2. Analysis of Free Energy Barrier

The sessile drop method reveals that contact angle hysteresis quantification is achievable through gradually varying the volume of the drop [12,33,34]. Consequently, this study employs an analysis of the free energy barrier variations during changes in drop volume to theoretically calculate the contact angle hysteresis. As the drop volume slowly decreases, both the constant contact radius mode and the constant contact angle mode can be experimentally observed [35]. When a receding drop loses a volume Δ*V* through the constant contact radius mode, the drop volume is altered to

(11) $$V_{\mathrm{pin}}^{\mathrm{rec}}=\frac{\pi r^{3}}{3\sin^{3}\left(\theta -\Delta \theta \right)}\left(\cos^{3}\left(\theta -\Delta \theta \right)-3\cos\left(\theta -\Delta \theta \right)+2\right)$$

where Δ*θ* is the change in the apparent contact angle. Conversely, when a receding drop loses a volume Δ*V* through the constant contact angle mode, the drop volume is altered to

(12) $$V_{\mathrm{depin}}^{\mathrm{rec}}=\frac{\pi {\left(r-\Delta r\right)}^{3}}{3\sin^{3}\theta }\left(\cos^{3}\theta -3\cos\theta +2\right)$$

where Δ*r* is the variation in the drop base radius, *r*.

Generally, a Wenzel drop can completely wet a rough surface structure; however, the study by Gao et al. has revealed that contact angle hysteresis is governed by the interaction of the drop boundary with local surface structures [27]. When contact lines advance or recede by a very small distance, the free energy barrier primarily depends on the system’s free energy changes and frictional tension [11,30]. As the contact lines start to recede, new solid–air interfaces are formed. Apparently, the sidewalls of the pillars at the drop boundary cannot be fully covered by the liquid [28]. Inspired by the definition of surface roughness, when the contact lines recede over the unit area on the projected surface, the increase in the air–solid interface area is estimated as (4α*ah* + *a*(*a* + *b*))/*a*(*a* + *b*), where α is a coefficient estimating the change in the air–solid interface of the pillar sidewalls. For different motion modes, the free energy difference in the system can be estimated as

(13) $$\Delta F_{w}^{\mathrm{rec}}=\left(\frac{2\pi {\left(r-\Delta r\right)}^{2}}{1+\cos\theta }-\frac{2\pi r^{2}}{1+\cos\left(\theta -\Delta \theta \right)}\right)\gamma_{\mathrm{la}}+\frac{4\alpha h+a+b}{a+b}\left(2\pi r\Delta r-\pi \Delta r^{2}\right)\left(\gamma_{\mathrm{sa}}-\gamma_{\mathrm{sl}}\right)$$

For a receding drop, the free energy barrier induced by frictional tension results from the increasing solid–air interface [11]. When contact lines recede by Δ*r*, the additional free energy barrier can be estimated as [(4*αah* + *a*(*a* + *b*))/*a*(*a* + *b*)](2*πr*Δ*r* − *π*Δ*r*^2^)*γ*_sa_. Thus, the receding free energy barrier is modified as

(14) $$\frac{F_{\mathrm{barr},w}^{\mathrm{rec}}}{\gamma_{\mathrm{la}}}=\frac{2\pi {\left(r-\Delta r\right)}^{2}}{1+\cos\theta }-\frac{2\pi r^{2}}{1+\cos\left(\theta -\Delta \theta \right)}+\frac{4\alpha h+a+b}{a+b}\left(2\pi r\Delta r-\pi \Delta r^{2}\right)\left(\cos\theta_{Y}+\frac{\gamma_{\mathrm{sa}}}{\gamma_{\mathrm{la}}}\right)$$

**Figure 3:**
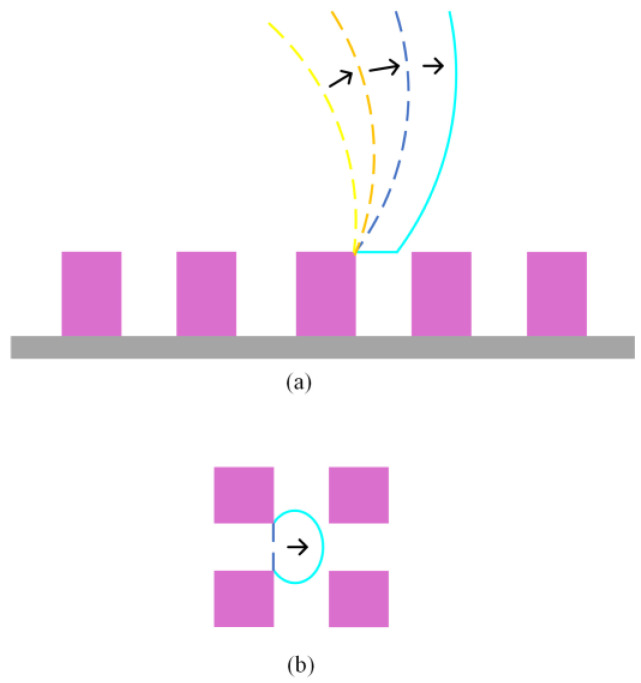
(**a**) A schematic diagram of the changes in the liquid–air interface above the pillars. (**b**) Advance of the contact lines between the pillars.

For an advancing Wenzel drop, the motion of contact lines is more complex. As the drop volume increases slowly, the apparent contact angle of the drop also increases gradually, as shown in Figure 3a. Once the apparent contact angle of the drop equals the advancing contact angle, for the contact lines on the tops of pillars (Figure 3a), experimental observations have shown that the detachment of contact lines can generate new air–liquid interfaces, and then the liquid gradually fills the surface structures [36]. The area of the generated air–liquid interfaces is written as (*a*/(*a* + *b*))(2*πr*Δ*r* + *π*Δ*r*^2^). Clearly, due to the complete contact between the pillared surface and the Wenzel drop, as the drop boundary advances, the liquid occupying the spaces between adjacent pillars also needs to move forward, as illustrated in Figure 3b. Consequently, contact lines also exist at the base of the pillar structures, specifically in the spaces between adjacent pillars. For the contact lines between the pillars, the advancing contact lines also induce an increase in the air–liquid interface (Figure 3b). Similar to the analysis of the increasing surface area of the air–solid interface for a receding drop, when the contact lines advance over the unit area on the projected surface, the generated air–liquid interface area is written as *βbh*/(*bd*), where *β* is a coefficient that corrects and estimates the area of the curved air–liquid interface. After the drop increases by a volume Δ*V*, the free energy difference in the system can be estimated as

(15) $$\begin{array}{c} \Delta F_{w}^{\mathrm{adv}}=\left(\frac{2\pi {\left(r+\Delta r\right)}^{2}}{1+\cos\theta }-\frac{2\pi r^{2}}{1+\cos\left(\theta +\Delta \theta \right)}\right)\gamma_{\mathrm{la}}+\left(2\pi r\Delta r+\pi \Delta r^{2}\right)\left(1-\frac{a}{a+b}\right)\left(\gamma_{\mathrm{sl}}-\gamma_{\mathrm{sa}}\right)\\ +\frac{a}{a+b}\left(2\pi r\Delta r+\pi \Delta r^{2}\right)\gamma_{\mathrm{la}}+\frac{\left(\beta -1\right)h}{a+b}\left(2\pi r\Delta r+\pi \Delta r^{2}\right)\left(1-\frac{a}{a+b}\right)\gamma_{\mathrm{la}} \end{array}$$

For the effect of frictional tension, the free energy barrier is attributed to the increasing solid–liquid interface [11] and is estimated as (2*πr*Δ*r* + *π*Δ*r*^2^)(1 − *a*/(*a* + *b*))*γ*_sl_. Accordingly, the advancing free energy barrier is modified as

(16) $$\begin{array}{c} \frac{F_{\mathrm{barr},w}^{\mathrm{adv}}}{\gamma_{\mathrm{la}}}=\frac{2\pi {\left(r+\Delta r\right)}^{2}}{1+\cos\theta }-\frac{2\pi r^{2}}{1+\cos\left(\theta +\Delta \theta \right)}-\left(2\pi r\Delta r+\pi \Delta r^{2}\right)\left(1-\frac{a}{a+b}\right)\left(\cos\theta_{Y}-\frac{\gamma_{\mathrm{sl}}}{\gamma_{\mathrm{la}}}\right)\\ +\left(2\pi r\Delta r+\pi \Delta r^{2}\right)\left(\frac{a}{a+b}+\frac{\left(\beta -1\right)h}{a+b}\left(1-\frac{a}{a+b}\right)\right) \end{array}$$

## 3. Results and Discussion

### 3.1. Analysis of Free Energy and Equilibrium Contact Angle

**Figure 4:**
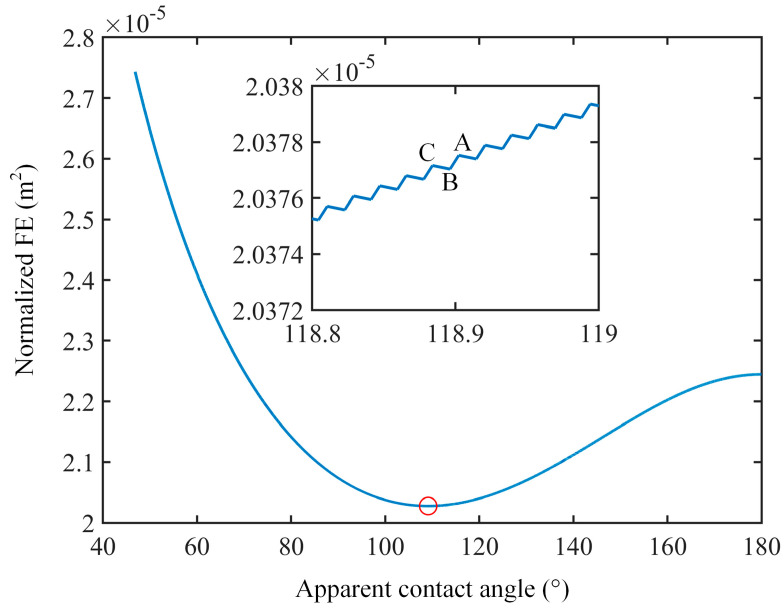
Normalized free energy versus apparent contact angle for Wenzel state. (*a* = 100 nm, *b* = 200 nm, *h* = 200 nm, *V* = 10 µL, and *θ*_Y_ = 100°).

To elucidate the equilibrium state and equilibrium contact angle of the system, Figure 4 presents the calculations of the free energy change for the Wenzel state as a function of the apparent contact angle. The surface with the dimensions *a* = 100 nm, *b* = 200 nm, and *h* = 200 nm is employed. As the contact line moves, the apparent contact angle gradually decreases from 180°, accompanied by significant changes in the system’s free energy. The minimum value of free energy on a global scale (indicated by empty circles) and multiple local free energy minima can be observed on the free energy curve for the Wenzel state, which determine the location of the contact line. As the contact line continues to advance through position A, B, and C (Figure 2b), a fluctuation is observed on free energy curves, indicating that the free energy evolves through a series of local free energy minima because of the different locations of the contact line. The contact line should be at position B, where the nanopillars are fully covered by the drop when the drop stops moving, because other positions, such as A or C, are not thermodynamically favored due to local free energy maxima, as shown in the enlarged drawing in Figure 4. Generally, the global free energy minimum corresponds to the equilibrium state, and the apparent contact angle at this state is defined as the equilibrium contact angle [37]. Consequently, the equilibrium contact angle is 109.1°, as indicated by the empty circles in Figure 4.

To validate the current approach, equilibrium contact angles for different pillar spacings and widths were calculated using the Wenzel equation and compared with the calculated results of the current model, as shown in Table 1. For varying pillar spacings and widths, the maximum error is less than 1°, indicating that the agreement between the equilibrium contact angles is calculated by different methods. Given that the Wenzel equation has been widely accepted by the majority of researchers [5,7,10,14,15], these results validate the effectiveness of the model in describing the free energy of the system.

### 3.2. Analysis of Free Energy Barrier and Contact Angle Hysteresis

In order to analyze the contact angle hysteresis of Wenzel drops, given the lack of experimental contact angle hysteresis data for nanostructured pillar surfaces in the current literature, a squarely arrayed square micropillar surface is used as a case study. The relevant parameters of the micropillared surface are adopted from the work of Forsberg et al. [28]: *a* is 20 µm; *b* is 150 µm; *h* is 7 µm. For the smooth substrate, the receding contact angle (*θ*_r_), Young’s contact angle (*θ*_Y_), and advancing contact angle (*θ*_a_) are 59°, 65°, and 72°, respectively [28]. The values of *γ*_sl_/*γ*_la_ and *γ*_sa_/*γ*_la_ are estimated using Equations (17) and (18), respectively [11].

(17) $$\cos\theta_{a}=\cos\theta_{Y}-\frac{\gamma_{\mathrm{sl}}}{\gamma_{\mathrm{la}}}$$

(18) $$\cos\theta_{r}=\cos\theta_{Y}+\frac{\gamma_{\mathrm{sa}}}{\gamma_{\mathrm{la}}}$$

**Figure 5:**
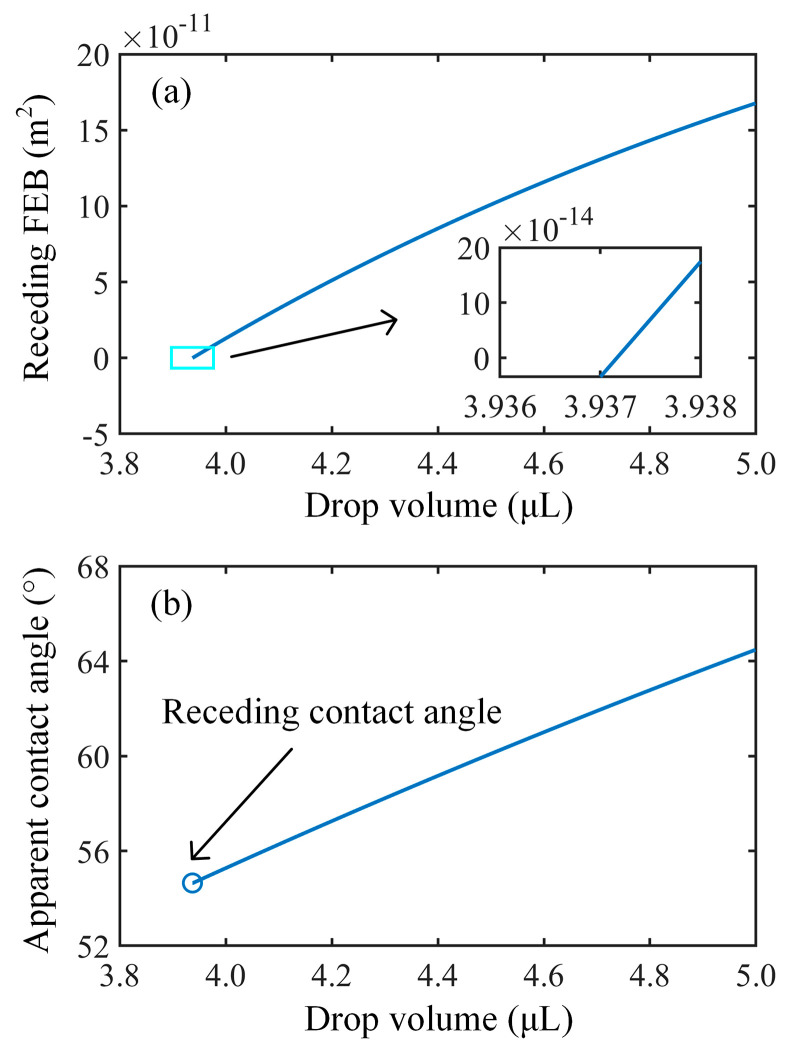
Variations in (**a**) normalized receding free energy barrier and (**b**) apparent contact angle with drop volume on a micropillared surface for Wenzel drops.

**Figure 6:**
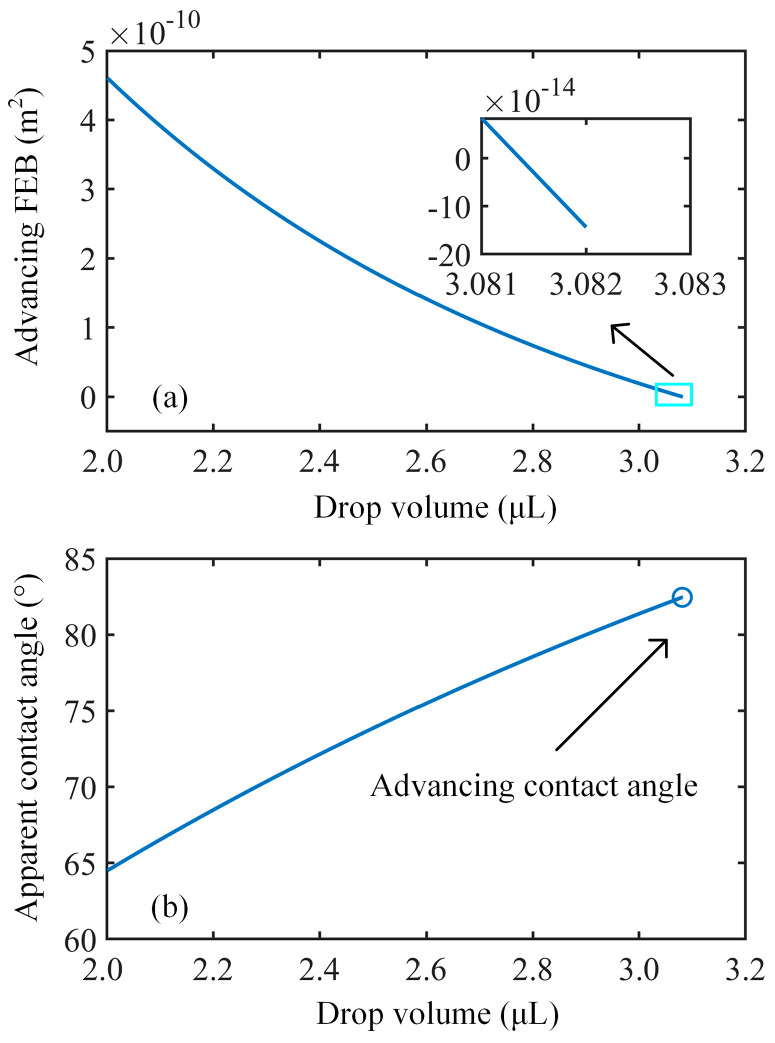
Variations in (**a**) normalized advancing free energy barrier and (**b**) apparent contact angle with drop volume on a micropillared surface for Wenzel drops.

The coefficients *α* and *β* are set to 0.75 and 1.65, respectively. Figure 5 illustrates a typical receding free energy barrier and apparent contact angle variation with respect to drop volume on the above-described micropillared surface. The receding free energy barrier shows a monotonic variation and decreases with decreasing drop volume, as shown in Figure 5a. At the critical drop volume of 3.937 µL, the receding free energy barrier becomes negative, and the motion of the contact lines becomes thermodynamically favored. Accordingly, the receding contact angle is determined to be 54.6° (Figure 5b). Figure 6 illustrates the advancing free energy barrier and apparent contact angle variations against drop volume. When the drop volume begins to increase, a positive advancing free energy barrier leads to pinned contact lines, bringing about the increasing apparent contact angle. At the critical drop volume of 3.082 µL, the motion of the contact lines is initiated by the negative advancing free energy barrier, and the advancing contact angle is quantified as 82.5°.

**Figure 7:**
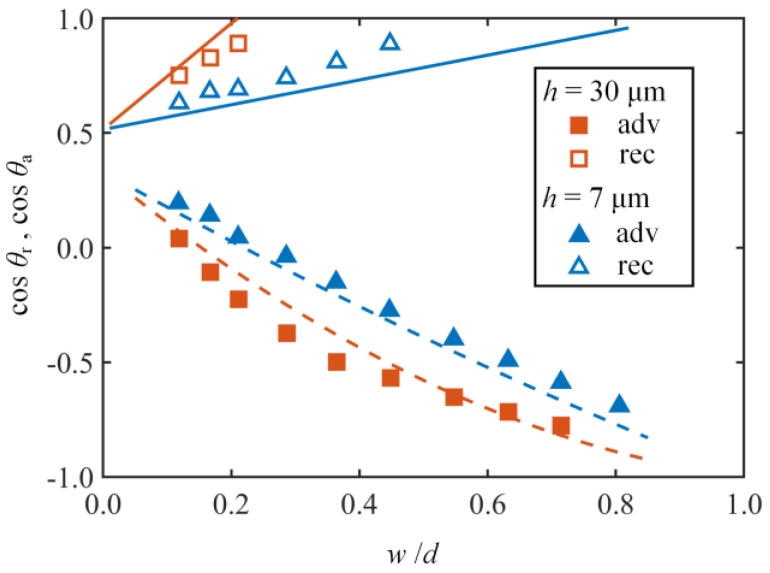
Predicted (lines) and measured (symbols) receding and advancing contact angles for pillared surfaces with different pillar heights (*h* = 7 µm, 30 µm) against the ratio of the pillar width *w* to the pitch *d*. The experimental values have been reported by Forsberg et al. [28].

To test the model’s validity, Figure 7 illustrates a comparison of experimental data for advancing and receding contact angles of Wenzel drops on micropillared surfaces with the various dimensions as reported by Forsberg et al. [28] with the values calculated by the model in this work. The pillar width *a* is fixed at 20 µm, and the pitch *d* (*d* = *a* + *b*) is used to control the pillar density *a*/*d*. The pillar height *h* is set to 7 µm and 30 µm. For shorter pillars (*h* = 7 µm), the advancing and receding contact angles exhibit linear behavior with respect to pillar density. In contrast, for taller pillars (*h* = 30 µm), the values of the advancing contact angles deviate from the linear relationship and move toward higher values. Apparently, the theoretical curves also follow this trend and fit the experimental data for different pillar heights and pillar densities.

Given the above thermodynamic analysis, it is evident that the interactions of the drop boundary and local structures of the solid surface significantly impact the contact angle hysteresis on the pillared surfaces for the Wenzel state, aligning with the theoretical analysis by Gao et al. [27]. As shown in Figure 7, although the theoretical curves can fit the experimental data, there are still some discrepancies between the theoretical and experimental results. These discrepancies primarily arise from the current limitations in calculating the changes in the system’s free energy. As previously discussed, the derivation of the free energy barrier necessitates modeling the variations in the system’s free energy. On the experimental side, the interface dynamics of receding and advancing Wenzel drops on rough surfaces have not been thoroughly visualized. Although the rheological characteristics of the Wenzel drop are expected to significantly impact contact angle hysteresis, the complete wetting of rough surfaces by the Wenzel drop presents substantial challenges for experimental design. The relationship between rheological characteristics of Wenzel drops and the contact line dynamics cannot be experimentally investigated. Additionally, the changes in the liquid–air interface caused by the motion of the contact line between micropillars and the contact line pinned on the tops of micropillars also require detailed experimental exploration. Moreover, precise experimental measurements of the changes in the liquid–air interface are also not currently feasible. Thus, the free energy change in the Wenzel wetting system is estimated approximately, incorporating the two correction coefficients *α* and *β* used in this study. Although the predicted results of the current model can aid in understanding the relationship between pillar structural parameters and contact angle hysteresis, experimental studies visualizing the Wenzel wetting system are necessary to better elucidate the related thermodynamic mechanisms and to reduce the discrepancies between theoretical predictions and experimental results in the future.

Additionally, during the measurement of contact angle hysteresis, external factors such as mechanical vibrations may overcome the free energy barriers, potentially resulting in measured contact angle hysteresis values that are lower than the theoretical values [12,38]. Clearly, if these external influencing factors can be quantified, the relationship between the free energy barriers and drop volume shown in Figure 5 and Figure 6 could enable theoretical predictions of contact angle hysteresis in the presence of external disturbances. This represents a potential future application of the current model.

## 4. Conclusions

This study introduces a thermodynamic methodology for the theoretical investigation of contact angle and contact angle hysteresis on pillared surfaces in the Wenzel state. The relationship between the free energy of the system and the apparent contact angle is quantitatively calculated, with the equilibrium contact angle determined by minimizing the system’s free energy. The equilibrium contact angles calculated using this model are consistent with the theoretical results obtained from the Wenzel equation. Regarding contact angle hysteresis, the thermodynamic calculations show that the free energy barrier decreases when liquid is incessantly being added to or is extracted from the drop, which governs the movement of the contact line. The apparent contact angles at which the receding and advancing free energy barriers become zero are identical to the receding and advancing contact angles, respectively. To assess the model’s accuracy, the predicted results are corroborated against experimental data from the literature, yielding a high degree of consistency. This model is expected to enhance the understanding of Wenzel drop movement on pillared surfaces.

## Figures and Tables

**Table 1:** Comparison of equilibrium contact angles calculated by the present model (*θ*_e_) with those predicted by the Wenzel equation (*θ*_w_).

Surface Structural Parameters			*θ*_e_(°)	*θ*_w_(°)
*a* (nm)	*b* (nm)	*h* (nm)		
100	100	100	110.3208	110.3220
100	200	100	104.5255	104.5263
100	300	100	102.5223	102.5364
100	400	100	101.6156	101.6207
100	100	200	121.3917	121.3956
200	100	200	118.8389	118.8394
300	100	200	115.7313	115.7293
400	100	200	113.3067	113.3232

## Data Availability

Data are contained within the article or Supplementary Materials.

## Supplementary Materials

The following supporting information can be downloaded at https://www.mdpi.com/article/10.3390/nano14231978/s1, Figure S1: Illustration of a drop wetting a pillared surface. (a) Initial placement of the drop on the surface. (b) Continuous advancement of the contact line; Figure S2: (a) Top view of the drop boundary moving from point A to point B. (b) Top view of the drop boundary moving from point B to point C.
