# Supplementary material for: Theoretical Analysis of Contact Angle and Contact Angle Hysteresis of Wenzel Drops on Superhydrophobic Surfaces
Source: Nanomaterials (Basel). 2024 Dec 9;14(23):1978. doi: 10.3390/nano14231978 (PMC11643717; doi:10.3390/nano14231978)
Supplement: Supplementary file 1 [file nanomaterials-14-01978-s001.zip › nanomaterials-3337290-supplementary.pdf]

*Supplementary information*

# Theoretical Analysis of Contact Angle and Contact Angle Hysteresis of Wenzel Drops on Superhydrophobic Surfaces

**Yufeng Li [1,*], Junyan Liu [1], Jialong Dong [1], Yufeng Du [1,2], Jinchun Han [1,3] and Yuanyuan Niu [1,*]**

[1] College of Electrical and Power Engineering, Taiyuan University of Technology, Taiyuan 030024, China; hanjc1998@163.com (J.H.)
[2] SEDIN Engineering Co., Ltd., Taiyuan 030000, China
[3] Inner Mongolia Electric Power (Group) Co., Ltd., Ordos Power Supply Branch, Ordos 017000, China
* Correspondence: li.yufeng@tyut.edu.cn (Y.L.); niuyuanyuan@tyut.edu.cn (Y.N.)

The derivation of some typical equations in the main text:
The system's free energy is described by the equation [29]:

$$F = \gamma_{sa}S_{sa} + \gamma_{sl}S_{sl} + \gamma_{la}S_{la} \tag{S1}$$

in which the interfacial tensions are denoted by $\gamma_{sa}$, $\gamma_{sl}$, and $\gamma_{la}$, corresponding to the solid-air, solid-liquid, and liquid-air interfaces, respectively. The surface areas associated with these interfaces are given by $S_{sa}$, $S_{sl}$, and $S_{la}$. A drop, upon deposition on the pillared surface, initially presents a spherical shape, and makes initial contact at position A, as shown in Figure S1a. To minimize the system's free energy, the contact line shifts its location and the drop's shape changes [30]. The thermodynamic analysis of the free energy for the Wenzel state, when a drop wets the surface, is as follows.

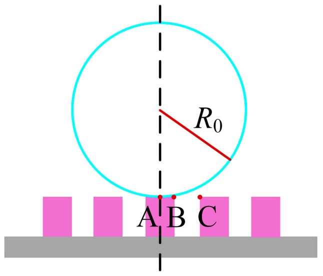


(a)

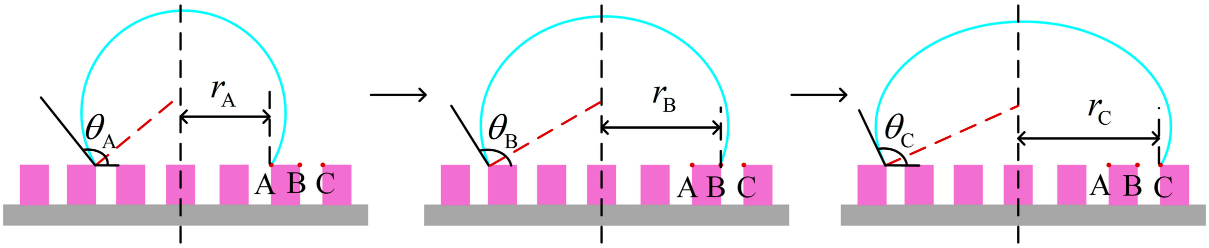


(b)

**Figure S1.** Illustration of a drop wetting a pillared surface. (a) Initial placement of the drop on the surface. (b) Continuous advancement of the contact line.

As shown in Equation (S1), the change in the system's free energy depends on the variation in the interfacial areas of various interfaces as the contact line moves. When a drop is initially placed on the material surface and makes contact at point A, the drop can be considered a sphere with a surface area given by $4\pi R_0^2$, where $R_0$ is the drop radius at position A. During the initial stage, as the contact line moves from position A to B (Figure S1a), the change in the liquid-air interfacial area is $2\pi r_B^2/(1+\cos\theta_B)-4\pi R_0^2$, while the changes in the solid-liquid and solid-air interfacial areas are represented by $a^2$. Considering these factors, the change in the system's free energy is given by:

$$\Delta F_{A\to B} = \gamma_{la}\left(\frac{2\pi r_B^2}{1+\cos\theta_B} - 4\pi R_0^2\right) - a^2\left(\gamma_{sa}-\gamma_{sl}\right) \quad \text{(S2a)}$$

Apparently, the free energy change can be simplified using Young's equation [31,32] and expressed as:

$$\begin{aligned}\frac{\Delta F_{A\to B}}{\gamma_{la}} &= \frac{2\pi r_B^2}{1+\cos\theta_B} - 4\pi R_0^2 - a^2\frac{\gamma_{sa}-\gamma_{sl}}{\gamma_{la}} \\ &= \frac{2\pi r_B^2}{1+\cos\theta_B} - 4\pi R_0^2 - a^2\cos\theta_Y\end{aligned} \quad \text{(S2b)}$$

where $\theta_Y$ is the intrinsic contact angle; $r_B$ and $\theta_B$ are the base radius and the apparent contact angle at position B, respectively.

In the Wenzel state, the liquid should fully wet the rough surface structure. Thus, as the drop boundary advances, the rough solid surface should gradually be wetted by the drop. As shown in Figure S1a, when the drop is first placed at point A on the material surface, it is spherical. Therefore, the drop volume can be calculated using the formula for the volume of a sphere, as shown in Equation (S3):

$$V_A = \frac{4}{3}\pi R_0^3 \quad \text{(S3)}$$

When the drop boundary advances to point B, as shown in Figure S1a, the drop is still only in contact with the top surface of the pillar, and the liquid has not filled the space between the pillars. Therefore, the drop volume can be calculated using the formula for the volume of a spherical cap, as shown in Equation (S4):

$$V_B = \frac{\pi r_B^3}{3\sin^3\theta_B}\left(\cos^3\theta_B - 3\cos\theta_B + 2\right) \quad \text{(S4)}$$

As the drop boundary moves to point C, as illustrated in Figure S1a, the Wenzel drop is in complete contact with the pillared surfaces, causing the drop to fill the space between the pillars from point B to C. Consequently, the drop volume is divided into two parts: the volume above the tops of pillars and the volume within the space between the pillars. The bottom of the drop can be considered a circle, so the volume of the drop contained in the space between the pillars from point B to C is given by $\left(\pi r_C^2 - a^2\right)h$. Therefore, the drop volume at point C can be expressed as:

$$V_C = \frac{\pi r_C^3}{3\sin^3\theta_C}\left(\cos^3\theta_C - 3\cos\theta_C + 2\right) + \left(\pi r_C^2 - a^2\right)h \tag{S5}$$

The pillared surface can be idealized as a repeating unit consisting of pillar spacing and width. Thus, the system's states at points A, B, and C (Figure S1b) represent a typical scenario where the drop boundary continuously moves through a series of pillar widths and spacings. Figures S1 and S2 correspond to the side view and top view of the drop boundary movement, respectively. When the drop boundary moves from point A to point B, as shown in Figure S1b and S2a, the drop volume is still composed of the volume above the pillar structure and the volume within the space between the pillars. To calculate the liquid volume within the space between the pillars, it is necessary to determine how many pillars exist beneath the drop. Assuming that the drop boundary can continue to move from point B to point C, as shown in Figure S2b—where the drop base radius changes from $r_B$ to $r_B + b$—the drop boundary can sweep over a complete repeating unit consisting of pillar spacing and width. Accordingly, the number of complete repeating units beneath the drop is $\pi\left(r_B+b\right)^2/\left(a+b\right)^2$, and the number of pillars beneath the drop is also $\pi\left(r_B+b\right)^2/\left(a+b\right)^2$. Thus, the volume of the drop at point B can be expressed as:

$$\begin{aligned} V_B &= \frac{\pi r_B^3}{3\sin^3\theta_B}\left(\cos^3\theta_B - 3\cos\theta_B + 2\right) + \pi r_B^2 h - \frac{\pi\left(r_B+b\right)^2}{\left(a+b\right)^2}a^2 h \\ &= \frac{\pi r_B^3}{3\sin^3\theta_B}\left(\cos^3\theta_B - 3\cos\theta_B + 2\right) + \pi h\left(r_B^2 - a^2\left(\frac{r_B+b}{a+b}\right)^2\right) \end{aligned} \tag{S6}$$

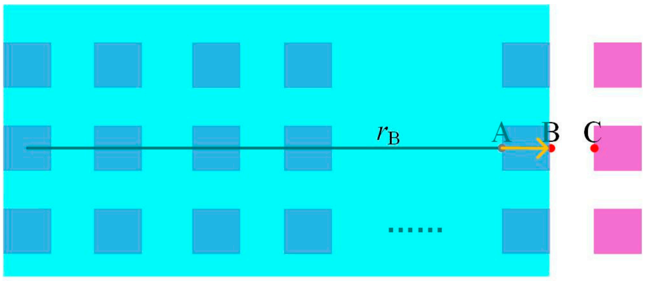


(a)

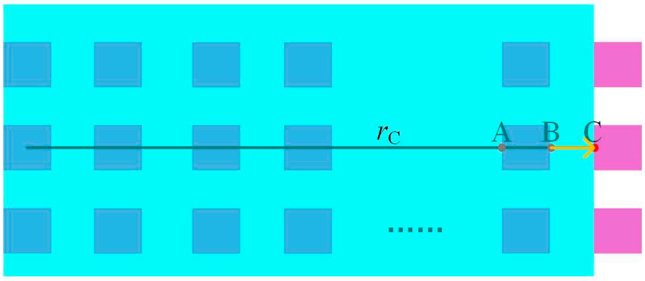


(b)

**Figure S2.** (a) Top view of the drop boundary moving from point A to point B. (b) Top view of the drop boundary moving from point B to point C.

As the drop boundary moves from point B to point C, as shown in Figure S2b, the boundary can sweep over a complete repeating unit consisting of pillar spacing and width. Accordingly, the number of complete repeating units beneath the drop is $\pi r_{\mathrm{C}}^{2} / (a+b)^{2}$. The number of pillars beneath the drop is also $\pi r_{\mathrm{C}}^{2} / (a+b)^{2}$. Therefore, the drop volume at point C can be expressed as:

$$
\begin{aligned}
V_{\mathrm{C}} &= \frac{\pi r_{\mathrm{C}}^{3}}{3\sin^{3}\theta_{\mathrm{C}}}\left(\cos^{3}\theta_{\mathrm{C}} - 3\cos\theta_{\mathrm{C}} + 2\right) + \pi r_{\mathrm{C}}^{2} h - \frac{\pi r_{\mathrm{C}}^{2}}{(a+b)^{2}} a^{2} h \\
&= \frac{\pi r_{\mathrm{C}}^{3}}{3\sin^{3}\theta_{\mathrm{C}}}\left(\cos^{3}\theta_{\mathrm{C}} - 3\cos\theta_{\mathrm{C}} + 2\right) + \pi r_{\mathrm{C}}^{2} h \left(\frac{b^{2} + 2ab}{(a+b)^{2}}\right)
\end{aligned}
\quad \text{(S7)}
$$

REFERENCES

29. Barbieri, L.; Wagner, E.; Hoffmann, P. Water Wetting Transition Parameters of Perfluorinated Substrates with Periodically Distributed Flat-Top Microscale Obstacles. *Langmuir* **2007**, *23*, 1723-1734, doi:10.1021/la0617964.

30. Li, W.; Amirfazli, A. Microtextured superhydrophobic surfaces: A thermodynamic analysis. *Adv. Colloid Interface Sci.* **2007**, *132*, 51-68, doi:10.1016/j.cis.2007.01.001.

31. Young, T. III. An essay on the cohesion of fluids. *Philos. Trans. R. Soc. London* **1805**, *95*, 65-87, doi:10.1098/rstl.1805.0005.

32. Dong, Z.; Levkin, P.A. 3D Microprinting of Super-Repellent Microstructures: Recent Developments, Challenges, and Opportunities. *Adv. Funct. Mater.* **2023**, *33*, 2213916, doi:10.1002/adfm.202213916.
